# Identification of human mutations in TRAF3IP1 in patients with nephronophthisis and retinal degeneration

**DOI:** 10.1186/2046-2530-4-S1-P52

**Published:** 2015-07-13

**Authors:** A Becker-Heck, A Bizet, R Ryan, P Krug, E Filhol, B Linghu, E Oakeley, F Serluca, F Legendre, N Dörner, MC Lasbennes, J Duca, F Yang, A Damask, L Klickstein, M Labow, M Schebesta, T Bouwmeester, H Valette, L Pinson, B Goubaux, P Dubot, R Salomon, C Antignac, M Gubler, C Jeanpierre, S Chibout, C Bole-Feysot, P Nitschké, A Benmerah, JD Szustakowski, AW Sailer, S Saunier, P Saint-Mezard

**Affiliations:** 1 NIBR, Novartis Institutes for Biomedical Research, Basel, Switzerland; 2 Laboratory of Hereditary Kidney Diseases, INSERM U1163, Hôpital Necker-Enfants Malades, Paris, France; 3 Institut Imagine, Université Paris Descartes Sorbonne Paris Cité, Paris, France; 4 Novartis Institutes for Biomedical Research, Cambridge, MA, USA; 5 Service de Pédiatrie, Centre Hospitalier Universitaire Arnaud de Villeneuve, Montpellier, France; 6 Service d'Anesthésie-Réanimation, Hôpital de L'Archet II, Centre Hospitalo-Universitaire de Nice, Nice, France; 7 Service de Néphrologie Pédiatrique, Hôpital Robert Debré, Paris, France

## 

Nephronophthisis (NPH) is an autosomal recessive inherited cystic kidney disorder. It represents the most frequent genetic cause of end-stage renal disease in the first three decades of life. NPH is characterized by the dysfunction of sensory cilia which explains the complexity of the NPH phenotype. It can be associated with retinitis pigmentosa (Senior-Løken syndrome), mental retardation and ataxia (Joubert syndrome), skeletal anomalies (Jeune syndrome), or *situs inversus*.

To date, recessive mutations causing NPH have been identified in more than eighteen different genes (NPHP1-NPHP18). Their gene products localize at the primary cilia-centrosome complex, along the cilium as intraflagellar transport proteins and are important in signaling pathways downstream of cilia including Wnt signaling, Shh signaling and the DNA damaged response pathway.

Using whole and targeted exome sequencing, we identified novel protein altering mutations in *TRAF3IP1 *in patients presenting with NPH, retinitis pigmentosa, skeletal defects of the pelvis, hexadactyly and hepatic fibrosis. *TRAF3IP1 *encodes IFT54 which is involved in the anterograde transport along the primary cilia.

Besides its known function in cilia we demonstrate that TRAF3IP1 act as a key regulator of cytoplasmic microtubule organization. Mass spectrometry analyses as well as pull-down experiments demonstrated that mutations in TRAF3IP1 lead to an altered binding to actin and microtubule associated proteins. Immunofluorescence stainings using patient fibroblasts as wells as mIMCD3 *TRAF3IP1 *knock-down cells confirmed the observed defects in microtubule organization. Furthermore, sphere formation assays as well as the pronephros of elipsa zebrafish embryos showed defects in epithelialization.

Altogether our findings demonstrate that NPH causing mutations of *TRAF3IP1 *affect both ciliary and non-ciliary functions of TRAF3IP1 which can provide an explanation for kidney tubules morphogenesis defects as well as the other disease phenotypes e.g. retinal, skeletal and hepatic defects.

